# Landscape governance as a matter of concern: A relational framework

**DOI:** 10.1007/s13280-025-02226-5

**Published:** 2025-08-09

**Authors:** Wim de Haas, Judith Westerink

**Affiliations:** Wageningen Environmental Research, Droevendaalsesteeg 3, 6708 PB Wageningen, The Netherlands

**Keywords:** Behaviour, Creativity, Ethics of care, Governance, Natality, Relational approach

## Abstract

In this article, we propose a relational social-ecological framework as alternative to functional views of nature, quantitative interpretations of value and bipolar conceptions of human–nature relationships. Instead, we conceptualise the complementary and reciprocal relations between nature, human behaviour and governance in a ‘landscape governance’ triangle in which: the relationship between nature and behaviour is conceptualised as signification and identity; the relationship between nature and governance as ordering and generation; and the relationship between governance and behaviour as authority and agency. From this relational conception, it implies that landscape governance is more a matter of concern than only a matter of fact, according to Bruno Latour’s proposal. We see this as the basis for an ethics of care that gives meaning to a relational social-ecological approach without losing the urgency of countering biodiversity loss and climate change. We illustrate our landscape governance triangle with examples from The Netherlands.

## Introduction

One of the great challenges of our time is the search for modes of inhabiting the earth while respecting natural conditions. In recent years, a broad range of concepts have been developed that should provide guidance in search for a kind of equilibrium between conservation and use, such as ecosystem services (De Groot et al. 2002; Cusens et al. 2023), rural viability (Zasada 2017), resilience (Walker et al. 2004), planetary boundaries (Rockström et al. 2009) and building on that, the doughnut economy (Raworth 2017). These concepts have inspired public policy, corporate responsibility initiatives and civic action. They have contributed greatly to more integrated sustainability approaches.

In several applications of this kind of concepts, however, we recognise some characteristic assumptions. First, a focus on quantification, mainly in order to strengthen the ecological aspect (quantitative thinking, see Dang et al. 2021). Second, a focus on functions, in which the usage or economic significance of nature for humans is central, even in applications that have a non-anthropocentric intent (functional thinking, see Dang et al. 2021). Third, a focus on contradictions between man–nature, object–subject and government–citizen (bipolar thinking, see Kadykalo et al. 2019).

It can be questioned whether this language of functionality, quantification and dichotomies as well as the underlying values contribute to a proper understanding of the complexity of socio-ecological systems, and have the potential to renew policies, practices and behaviours to the extent that environmental crises can be countered (Titeux et al. 2019; IPBES 2021). Several times in our research we have felt hampered by the limitations of this language. To express the nuances we seek, we propose a relational framework for understanding the mutual interaction between nature, human behaviour and governance An important choice in this framework is the focus on landscapes as being both social and ecological (Herlin 2004; Görg 2007; Arts et al. 2017). In the framework, landscape is more than the quantitative aspect of the environment, transcends the opposition between humans and environment and is not limited to the functions of the environment. In our framework, we also give more depth to the relational character of the concept of ‘landscape governance’, that covers the type of processes that can respect natural conditions and can connect governance of the physical landscape with values and identities of the people that make and use this landscape (Görg 2007; Gonçalves and Pinho 2024).

The relational tradition of sustainability research focuses on the relationships and connections rather than on the entities of the social-ecological system (Swyngedouw and Heynen 2003; Latour 2005; Stenseke 2018; West et al. 2020). Relational thinking implies that the existence and meaning of entities, such as actors (farmers, governments, firms), groups or ecosystems, are determined by their interrelationships. Moreover, relational thinking acknowledges that (meanings of) things and relations are socially constructed, that they are dynamic and that they are interdependent.

In line with West et al. (2018) and West et al. (2020) who identify care as an important component of relational thinking, our framework is based on the normative idea of care, which we consider to be an essential feature of human existence. Care can be defined as “a species of activity that includes everything we do to maintain, contain and repair our ‘world’ so that we can live in it as well as possible. That world includes our bodies, ourselves and our environment” (Fisher and Tronto 1990). As a consequence, we conceptualise the relationships between nature, behaviour and governance not as relationships based on utility or duty, but based on care. In a care relationship, instead of mutual self-interest, the quality of the relationship is the primary concern. We will argue that the idea of care is expressed in an approach of landscape governance as a matter of concern rather than a matter of fact.

After describing the framework, we illustrate it with two examples from the Netherlands. These examples draw on previous research by the authors. The first example is based on qualitative research into social conflicts about the type of nature management (De Haas et al. 2019, 2020). This study was aimed to distinguish some characteristic interaction patterns. This was conducted in two different areas through 14 semi-structured interviews with actors selected on basis of their role in the conflict. The second example is based on studies on the role of agri-environmental collectives in landscape management (Westerink et al. 2020b, 2021). For this qualitative study, a total of 56 interviews were conducted with farmers, representatives of farmer groups and other stakeholders (67 persons in total) throughout the country. We conclude with a reflection on possible action perspectives.

## Theoretical framework

In this section, step by step and building on the literature, we propose a framework for understanding the relations between nature, (human) behaviour and governance. As point of departure, we take the concept of ‘landscape governance’ because of its relational qualities as place-based, inclusive and social-ecological, bringing together the concepts of nature, behaviour and governance.

### Landscape governance—connecting nature, behaviour and governance

We understand landscape as the interplay between natural processes and human behaviour, and it therefore has natural-material aspects as well as socio-cultural aspects in a specific spatial context (in line with the European Landscape Convention: Council of Europe 2000). The concept of landscape governance was introduced by Görg (2007) as grounding environmental governance in specific spatial contexts with specific biophysical conditions and acknowledging the relations between people and nature and social construction of place. Buizer et al. (2016) operationalise landscape governance as ‘the interplay of discourses, institutional practices and natural-spatial conditions’. Both Görg and Buizer et al. stress the political aspects of the construction of scale, which often results in deviating power away from the people who ‘make’ and ‘use’ the landscape. In contrast, landscape governance is based on plurality of landscape comprehensions, interdisciplinarity and multi-actor collaboration (Westerink et al. 2017b; Ros-Tonen et al. 2018; van Oosten et al. 2018; de Koning et al. 2023). Building on this literature, we elaborate on the relational aspects of landscape governance. Before conceptualising the relations between nature, human behaviour and governance in "Reciprocal and complementary relations" section, here we first briefly introduce these three concepts.

*Nature* can be understood in many ways, for example as a physical process, biodiversity, a service or a value. These are all similarly ‘difficult’ and comprehensive concepts with a great diversity of interpretations, based on a variety of worldviews. Pascual et al. (2021) describe an inclusive typology that classifies nature values by level of abstraction and by worldview. Different worldviews at the highest level of abstraction lead to different indicators of the value of nature at the most concrete level. The relational framework we develop here is in line with this. When we speak about ‘nature’, we acknowledge that our perceptions of nature are strongly influenced by our relation to nature and vice versa. The definition of nature emerges from the nature–behaviour relationship and the nature–governance relationship. A purely functional relationship leads to a different conception of nature than a care relationship (see below). This conceptualisation makes our framework inclusive for a great diversity of meanings, which should be acknowledged to be able to recognise and address challenges in the human–nature relation (Pascual et al. 2021).

*Behaviour* describes what people do, deliberately or based on impulse, routinely or in reaction to circumstances, and often in response to their social environment (Ajzen 1991; Stryker and Burke 2000; Vatn 2005; Thaler and Sunstein 2008). In this article, we particularly consider behaviour affecting the natural environment, such as agricultural practices, tourism and eco-activism. The most basic human behaviour regarding nature is related to bodily and psychological necessity, such as the acquisition of food or recreation. Also, human activity can take the form of protection, restoration or worship, driven by values and beliefs. However, often it is driven by social and economic processes in which people become so detached from nature that over-exploitation or destruction become likely (Swanwick 2009). A range of factors has been identified that influence environmental behaviour and that can therefore also function as a barrier to behaving responsibly (Kollmuss and Agyeman 2002).

*Governance*. While behaviour refers to practices and experiences of people, governance refers to (arrangements for) collective action. Governance has an important role in reproduction or modification of conditions for behaviour (Giddens 1984). The governance concept, better than policy, captures the idea that not only governmental actors steer developments in society, but a wide range of actors is steering actively and influence each other in complex governance networks. Traditionally, governance has been understood as a task of governments who can use legal, economic and physical means in order to enforce or encourage a pursued behaviour. However, under influence of processes such as globalisation, regionalisation and informatisation, there has been a shift from ‘government' to ‘governance’ (Driessen et al. 2012). This is a collaborative style of collective action, which recognises a policy role of market players and civil society actors in addition to that of the government. Governance therefore does not coincide with governmental action, although the government does have a specific role, based on the exclusive use of certain instruments to influence behaviour.

Clearly, nature, behaviour and governance are closely related: they respond to each other and are interdependent. They all come together in the landscape.

### Matters of concern and ethics of care

As pointed out in the introduction, we are looking for a perspective that does not imply antagonisms between humans and nature, hierarchical relationships between citizens and governments or singular definitions of nature, only as given facts. Such assumptions may suggest certainty, but contribute to a view of reality that characterise alternative views on phenomena as irrelevant or unrealistic. Latour (2004) would phrase such assumptions as ‘matters of fact’. In this way of thinking, facts are understood as standardised fixed images of states of affairs.

Latour also describes the downsides of this line of reasoning. If we approach reality as a matter of facts, that is, if we accept facts just because they are considered as facts, we become blind to new phenomena, to the unknown or to contradictions. In addition, viewing phenomena in terms of facts neglects the normative foundations of concepts and their value to people, which impoverishes debates and excludes viewpoints. In this way, facts narrow the scope of our perception. Those who want to escape this, who do not feel heard but seek recognition for their viewpoints, sometimes seek comfort in so called ‘alternative facts’. However, such a ‘fact-free’ approach undermines the whole concept of factuality. This is why Latour argues for a broader perspective: a perspective that considers reality as a *matter of concern*. When reality is approached as a matter of concern, all aspects of perception and experience are mobilised from a deep interest in, engagement with and concern for the phenomenon in question, which can also lead to accepting parts of reality as established fact. This concern prevents that relational thinking becomes relativist thinking, for example in a conclusion that the loss of biodiversity is not important because all facts are socially constructed. But at the same time this concern opens the option to reason not only on the basis of facts but also on the basis of (relational) values.

The perspective that regards reality as a matter of concern is linked to an ethics of care (Tronto 1993). The ethics of care seeks its principles for righteous behaviour not in the usefulness of actions or in moral obligations, but in caring as intrinsic to existence. This implies an appeal to be sensitive to the differences in our complex environment, and a focus on the nature of the object of care. This sensitivity is very similar to what Latour calls concern. Caring is a practice in which experiences are more important than duties, rules or utility. Caring implies: the recognition of needs, the responsibility for the identified needs, the actual care-giving and the responding to the received care (Tronto 1993). Latour’s conceptualisation of concern and Tronto’s notion of care are aligned: one could argue that care is the practice that conceives its field of action as a matter of concern.

### Reciprocal and complementary relations

**Fig. 1:**
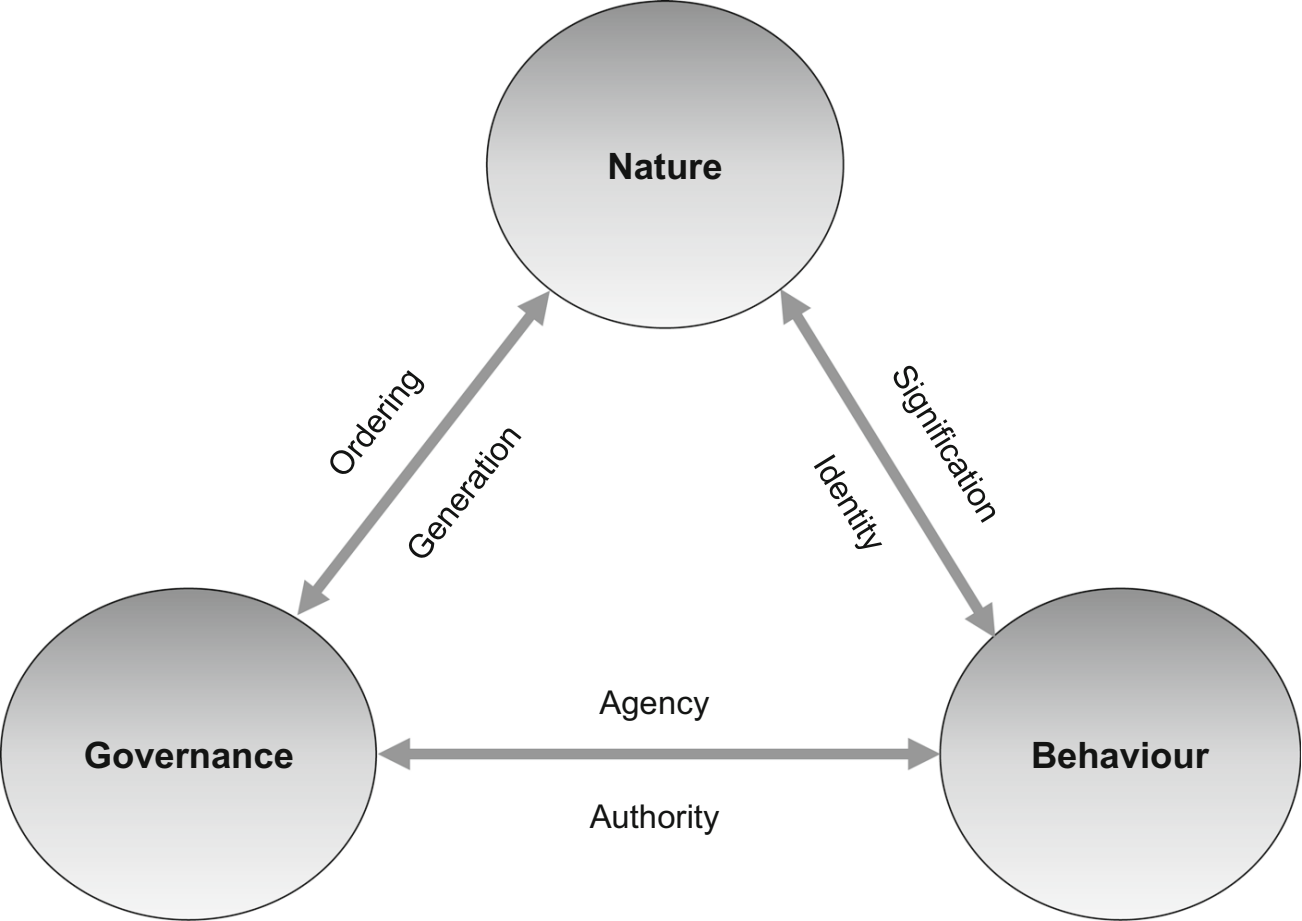
Landscape governance triangle: reciprocal and complementary relations between nature, behaviour and governance

Visualising landscape governance as a triangle relating nature, behaviour and governance (Fig. 1) invites further consideration of the relationship between these three. In this triangle, we propose reciprocal and complementary relations between nature, behaviour and governance. With reciprocal we mean that the relation goes both ways: nature influences behaviour, and behaviour influences nature, the same applies, mutatis mutandis, for the other relations. With complementarity, we refer to every relationship having two aspects that complement each other, like two sides of a coin. In the following, we will first conceptualise these complementary relations, and then characterise them in a broader context.

#### Nature and behaviour (signification and identity)

Nature has a broad range of meanings for people, ranging from threat, source of food, wealth and enjoyment to sense-making and spirituality. Therefore, meaning is an important aspect of the relation between behaviour and nature. We argued that definitions of nature are determined by the relation between nature and behaviour. Conversely, behaviour is also determined by the meaning we give to nature; meaning shapes both the perception of nature and the mode of behaviour. Or, according to Keulartz et al. (2004) *‘concepts of nature form complicated value structures made up of cognitive, normative and expressive elements. They must at the same time provide answers to the questions how reality should be scientifically represented, ethically judged, and aesthetically experienced.’* This notion is also reflected in theories of behaviour such as the Value Belief Norm Theory and the Theory of Planned Behaviour, which recognise a strong link between attitudes, values, convictions, norms and identity and behaviour of people, including environmental behaviour (Ajzen 1991; Stern 2000; Johansson et al. 2013; Horlings 2015; Wauters et al. 2017). Further, symbolic interactionism states that things, including nature and our own identity, get meaning through interactions with those things and with other people (van Kraalingen and Beames 2024). To denote the emergence of symbolic meanings that people attach to nature or to elements in the landscape, which pervades both the concepts of nature and human behaviour, we propose the term *signification* (see also (Gustavsson 2012).

In addition, the nature–behaviour relationship includes conceptions of human *identity* in relation to nature: for example as enemy, master, steward, partner, friend or component of nature. Such identity concepts relate to behaviours because people prefer to see themselves as behaving in congruence with their self-image: doing things that their kind of people would do (Lokhorst et al. 2011; Hosany and Martin 2012). Identity has multiple layers. Identity can relate to certain places which are important to the individual (Creswell 2004). This is also called place identity (Peng et al. 2020) or existential identity (Mansvelt and Pedroli 2003). Individual identity, group identity and place identity all play a role in the way we personalise issues such as biodiversity loss and in subsequent pro-environmental behaviour (Udall et al. 2020).

*Signification* and *identity* are complementary aspects of the two-sided relationship between nature and behaviour. They are complementary because signification emerges from a certain identity and, conversely, identity is produced and reproduced in the process of signification.

#### Governance and nature (ordering and generation)

Next, we elaborate on the two aspects of the reciprocity between governance and nature. Governance is engaged with nature through various forms of collective action, but at the same time, it is the result of natural conditions in terms of ‘the behaviour’ of ecosystems and species.

Regardless of the extent of the governance intervention, the relationship between governance and nature always involves the creation of categories of place, land use and rights, including changes therein. Consequently, we define this aspect of the relationship between government and nature as *‘ordering’*, i.e. the creation of a specific order, whether it is comprehensive or partial.

In addition to the ordering aspect, the relation between nature and governance can also be characterised as *generative*. Nature can inspire or necessitate collective action to face threats from nature or to use the opportunities that nature offers. In these cases, it can be argued that nature generates governance. The Netherlands is often cited as an example, where the threat of flooding is said to be the cause of a tradition of collaboration and corresponding structures of governance. Conversely, governance also generates nature as a value by articulating nature conservation as a collective task.

We consider ordering and generation as complementary because they presuppose each other: creating order needs the emergence of collective goals (for example in relation to the generative qualities of nature), while a collective goal needs to manifest itself in a form of ordering.

#### Governance and behaviour (authority and agency)

When exploring the influence of governance on behaviour, it is important to keep in mind that actors are not only objects of governance, they are also part of it. It explains, for example, why policies based on a strict distinction between subject and object of policy often fail to succeed. The ‘performance’ of governance is expressed in qualities such as ‘good governance’, ‘governmentality’ and ‘governance capacity’ (Arts and Goverde 2006; Rose et al. 2006; Hartmann and Spit 2015). Other relevant literature investigates factors that influence behaviour, categorised as willingness, ability and opportunity or support, on which incentives could be tailored (Michie et al. 2011; Westerink et al. 2020a). Particularly in New Institutional Economics the impact of institutions, policies and instruments on behaviour are theorised (Williamson 2000; Vatn 2005).

We propose that governance and behaviour are related through processes of *authority* and *agency*. We understand both authority and agency as two-way relationships: it is not only the ‘sending’ done by the governing actor(s), but also the ‘receiving’ by the actor(s) whose behaviour is being influenced (Raz 2006; Lopes 2020). Authority indicates whether the message as well as the messenger of governance are worth listening to. Authority depends on whether the reasons for the required behaviour change as well as the required behaviour itself are considered fair and legitimate (Raz 2006; Bernstein 2011; Lopes 2020). In addition, authority indicates whether the governing actor is considered trustworthy and reliable (Raz 2006; Lopes 2020). Authority therefore is in the eye of the beholder. Whether governance influences behaviour depends both on the governing and on the governed actor.

Agency of such governed actors, or their power to take action, is determined by enablement and constraint as experienced by the reflective agent (Giddens 1984). For the agent to be able to change behaviour, the required behaviour needs to be enabled, and constraint needs to be reduced. Agency is therefore conditional for governance to succeed. To do so, governance should be targeted at strengthening enablement and reducing constraint in the context in which the actor operates. However, it is the reflective agent who does or does not change behaviour in response to the changed conditions. As argued above, this behaviour change is more likely when the agent acknowledges this as fair and legitimate and sees the governing actor as trustworthy and reliable. Therefore, because of this reflexivity, agency and authority are related (Lopes 2020).

#### Summarising the relations

These complementary and reciprocal relations manifest themselves as ‘matters of fact’ in a different way than as ‘matters of concern’. These different points of departure result in different types of governance of landscapes. In Table 1, we illustrate how this could play out. While in contemporary governance landscapes are often considered as matters of fact, we would define landscape governance as a matter of concern.

**Table 1:** Comparison between governance of landscapes as a ‘matter of fact’ and landscape governance as a ‘matter of concern’

Reciprocal relation	Complementary aspects	Governance of landscapes as a matter of fact	Landscape governance as a matter of concern
Nature and governance	Ordering	Distribution of rights of access to and use of nature, land use planning. Nature policy is aimed at nature categories	Respecting natural conditions, protection of valuable and vulnerable landscapes. Nature policy focuses on relationships with nature
	Generation	Nature as resource, providing goods and services to humans. Focus on production and monetary value	Nature as a source, generating what humans need for living. Focus on regeneration and community value
Governance and behaviour	Authority	Hierarchical governance based on regulations and scientific knowledge	Aligning messages about desirable behaviour with values of people who make and use the landscape. Authority of local, informal leaders
	Agency	Enabling agency of formal nature organisations and land owners, constraining agency of bottom-up initiatives	Enabling caring agency, constraining destructive agency
Behaviour and nature	Signification	Reproducing objectified images of nature and a nature-human dichotomy	Facilitating the construction of meanings and symbols that signify belonging, ownership, good craftsmanship, sharing and a positive human–nature relationship
	Identity	Reproducing identity concepts of humans as consumers, masters or enemies of nature	Stimulating identity concepts of humans as stewards, partners or part of nature

**Fig. 2:**
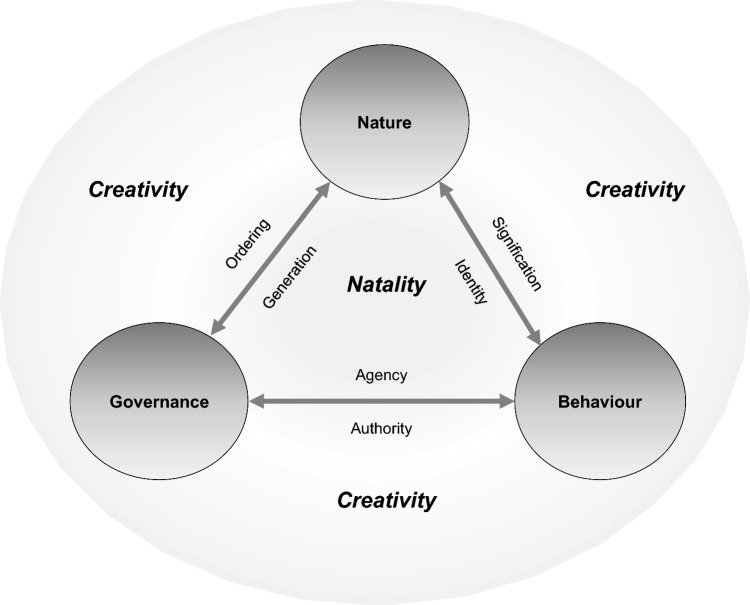
Landscape Governance Triangle: between natality and creativity

As said, the three relationships between nature, behaviour and governance are not only reciprocal but also have two complementary sides. We visualised identity, agency and generation on the internal side of the landscape governance triangle, and signification, authority and ordering on the external side (Fig. 2). For the characterisation these sides, we were inspired by Hannah Arendt’s concepts of natality and creativity. *Natality* is the capability to start or initiate something new (Arendt 1958). According to Arendt, like mortality, natality is a basic condition of being. Arendt places herself in opposition to those who see the end (death) as the most essential characteristic of existence. Birth is not only the principle of starting anew, but also the principle of uniqueness. The three aspects on the inside of the landscape governance triangle, generation, agency and identity, all represent natality. *Generation* is the coming about of something unique, while *agency* being intentional refers to the birth of actions, and *identity* includes the views and beliefs from which actions are initiated. While the internal side of the landscape governance triangle represents the sources of renewal as types of natality, the external side stands for the names, forms and directions by which the relationships between nature, behaviour and governance take shape. We therefore characterise the external side with the concept of *creativity*. Ordering, authority and signification all imply ‘authorship’, and shape and communicate ideas and categories: these are processes of creation (Runco and Chand 1995; Ravet 2016). Arendt (1958) relates creativity to power. Power is necessary to create something—such as a new way of farming, or better care for the environment—and it arises where people do this collectively.

## Examples

In this section, we illustrate the landscape governance triangle with two examples. The first example accounts of contemporary nature policy conflicting with values of local stakeholders, in spite of a shared nature conservation commitment. The second example shows how shifting responsibilities for agri-environmental governance from the government to groups of farmers aligned better with and influenced values of farmers. Both examples suggest that forms of landscape governance can enhance a sense of ownership and care for nature with local stakeholders.

### Conflicts on nature management in the Netherlands

A common source of conflicts on nature management in the Netherlands is the question which kind of nature is to be conserved. Two examples are: the transformation of areas with forests to a previous ecosystem with many open spaces and native deciduous trees, and the hunting of large mammals (deer, roe deer, wild boar) in nature reserves where their grazing pressure negatively affects other nature goals than wildlife (natural regeneration of forest, species-rich grasslands).

The cutting of trees and hunting as forms of nature management have been regularly opposed by groups of citizens—for a typology of the different forms of resistance, see De Haas et al. (2020). For example, in Salland (in the east of the Netherlands) and in a dune area at the northwest coast residents protested fiercely against the cutting of trees. They received much support from all parts of society (De Haas et al. 2019). ‘Why should we cut trees in times of climate change?’ is one of their arguments.

In other parts of the Netherlands, the Oostvaardersplassen and the dune area of the province of South Holland, there were numerous protests against the shooting of large mammals and against the hunt of fallow deer. In both areas, protest meetings were organised, lawsuits were started, lobbies were targeted at politicians and social media were used extensively. The protest went so far that employees of nature organisations were physically threatened. Even bomb threats were made that ultimately turned out to be false.

In these conflicts, a tension emerged between the implementing governments and nature organisations on the one hand and local citizens on the other. Both sides received support from other parts of society. This tension raised despite various procedures for public participation. It appears that the issues of concern (as experienced by the citizens) were not addressed adequately in these procedures. The issues of concern raised by activist citizens are often based on deep sense of commitment to the current shape of nature: coniferous forest and large herds of mammals. These feelings are not acknowledged by governments because they are considered as non-rational. Meanwhile, the authorities base their authority on rationally elaborated choices (*ordering*). Furthermore, the impairment of authority is related to a time lag between decision-making and implementation. For most citizens, decisions about nature only become visible in the phase of implementation. Their opinions are not welcome at that stage of the process. In addition, communication by the government in these processes is mainly aimed at providing information and not at the exchange of ideas. As a result, citizens do not feel respected as agents. In short, the *authority aspect* of the governance–behaviour relationship is compromised and the *agency aspect* undermined because both the argumentation and the process of working are founded on rationality.

Against the backdrop of this general picture, mostly in response to the wide-spread protests, alternative developments emerged. In one specific case in the dune area, after several protests, accessible meetings organised by the local forest ranger contributed to support and understanding. The forest ranger concluded that he had to move from an attitude focussed on choices and factuality to an attitude focussed on the relationship with the protesting residents. This earned him respect and reinforced his authority. The protesting citizens felt more recognised as valuable agents. This resulted in a further participative detailing of (parts of) plans. Here, the acknowledgement of *agency* restored *authority*. It became clear that this is not a one-time action, but needs continuous attention, requiring time and budget.

In Salland, there was a similar situation: a forest that was highly valued by residents was to be cut down in favour of the restoration of heather, a more open semi-natural landscape. There were protests by some well-informed citizens. Recreational entrepreneurs also opposed the plans. The protesting citizens turned out to have a completely different relationship to nature and to use a different concept of nature than other nature-loving citizens. An action emerged that made it possible to conceive the forest not as a matter of fact but as a matter of concern, which diminished the contradiction. As remembered by residents, in the Second World War, in a certain part of the area there was a shelter where people were in hiding. This memory was part of the *identity* of local inhabitants and gave this area a specific *signification*, which was neglected in the original plans. In response to this story, part of the trees was felled to make the shelter visible again, while in another part the trees were spared. This action strengthened the *signification* of this area.

In these contested nature areas, governance is manifested in a planning (*ordering*) perspective on nature. This implies a choice of nature goals (established by Natura 2000), drawing up nature management plans and measures (like cutting down trees to create open areas) to implement these plans and to realise a new form of order. Many of these plans have a rational and quantitative character in which nature is seen as an object and as a non-human system. This ordering aspect of the relationship between government and nature dominates the generative aspect: detailed, substantive ecological knowledge was central to the planning. The idea of nature as inspiring source for collective responsibility and action (*identity*, *generation*) played only a limited role.

### Collective agri-environmental management and ‘good farming’

In the Netherlands, agri-environmental policy has long been shaped by subsidy schemes connecting payments to farmers to compensate for loss of production (*generation*) to detailed prescriptions for landscape management in specific areas, aimed at the protection of specific species (*ordering*). These prescriptions were based more on ecological expert knowledge than on knowledge of the farming system and practical knowledge of farmers. Policy makers assumed that scientific knowledge would provide the *authority* to make farmers accept the agri-environmental measures as sensible and desirable, and that the subsidies would strengthen farmers’ *agency* by making the measures economically feasible. However, participation levels remained low and the agri-environmental scheme was labelled ineffective (RLI 2013). It appeared that participating farmers either were intrinsically motivated (*identity*) or made the best of suboptimal landscape conditions by bringing their least productive landscape features under agri-environmental management (cf. Van Herzele et al. 2013). Many non-participating farmers however must have felt a conflict between agri-environmental management and their preference for ‘tidy landscapes’ as a display of their skill as a ‘good farmer’ (*signification and identity*) (cf. Burton et al. 2008). According to the ‘good farming’ literature (e.g. Burton 2004, 2012), farmers find their self-identity in their skills as a farmer which they display in the landscape. Producing ‘tidy landscapes’ with straight lines, evenly growing crops and no weeds is a way to show to their peers that they are good farmers. While moving through the landscape, farmers review each other’s land and they are aware that the others review their land as well. This way, cultural norms are reproduced and communicated that represent values of productivity and order (*generation and ordering*). Nature, in the form of weeds or ‘untidy’ landscape elements may be at conflict with the values and identity of a ‘good farmer’. This may explain low interest among farmers in participation in agri-environment schemes or organic farming (Burton et al. 2008; Sutherland and Darnhofer 2012). However, farming culture is not homogeneous or static and views have been reported in which biodiversity is appreciated on farmland as part of ‘good farming’, mainly after experience with biodiversity-friendly practices (Riley 2016; Cusworth 2020).

As of 2016, regional agri-environmental collectives have a major role in the implementation of the Dutch agri-environment scheme, based on the earlier experience with local environmental cooperatives (Westerink et al. 2020b). These farmer groups recruit and train participants (*authority and agency*), spatially coordinate the measures (*ordering*), make contracts with individual farmers, perform control and sanctioning (*authority*) and supply the payment (*agency*) (Westerink et al. 2017a). In addition, the collectives negotiate conditions of the scheme with the province and influence the design of the scheme as a whole. By 2021, the number of hectares under the scheme as well as the number of participating farmers have grown, while the ecological ambition of the contracts was raised (BoerenNatuur 2021, pp. 30–31, 35–36). As recruitment by the collectives yields more participants than before, collectives seem to have more *authority* with farmers than the government: as farmer group native to the region and knowledgeable of the landscape and its farming systems, the collective is often regarded as ‘our own’. In order to strengthen farmers’ *agency*, the collective not only provides payments for agri-environmental management, but also training and knowledge exchange among farmers. In turn, this strengthens the *authority* of the collectives. In addition, as a group of farmers skilled in agri-environmental management, they build a subculture in which a biodiverse landscape is a sign of ‘good farming’ (*signification*) and agri-environmental management belongs to the *identity* of a ‘good farmer’ (Westerink et al. 2021). Care is an essential part of the values of a ‘good farmer’, who takes good care of land and livestock. Under the previous agri-environment scheme, the management prescriptions (*ordering*) were often interpreted by farmers as ‘neglecting the land’. However, farmers, who have learned to recognise agri-environmental management on each other’s land (*signification*), can appreciate this as ‘good care’ because this land is not only destined to produce food, but also biodiversity (*generation*) (Westerink et al. 2021). Apparently, more than the government in the past, collectives manage connect to the relation between a farmer and his or her landscape at the level of signification and identity. Collectives are so effective at this that they contribute to cultural change (ibid).

### Comparison of examples

The examples are summarised and compared in Table 2. The examples illustrate that landscape governance as a ‘matter of concern’ implies a more relational type of governance. It became clear that hierarchical, technocratic governance, viewing the relationships between nature, behaviour and governance as matters of fact, fails to emancipate the caring values in society, which is a huge opportunity missed for integrating nature into our lives. In both examples, viewing nature as a ‘matter of concern’ enabled a sense of partnership with nature, and confirmed identities reflecting a positive human–nature relationship. Landscape governance as a matter of concern put ethics of care central: it spotlighted relational, caring values in the relations between behaviour and nature, it enabled more concern in the relation between governance and behaviour, and it brought more collectiveness in the relation between governance and nature. This is in line with findings of Klain et al. (2017) that relational values of stewardship, community, identity, responsibility, care and kinship are important in how people perceive ‘good life’ and their relation to nature.

**Table 2:** Summary of the examples

Relation	Characterisation of the relationship	Example nature management conflicts		Example collective agri-environmental management	
		Matter of fact	Matter of concern	Matter of fact	Matter of concern
Nature and governance	Ordering	Desired futures are presented as factual description of original unspoiled nature	Nature is considered as landscape	Agri-environment scheme with detailed management prescriptions aimed at specific species	Management is tailored to the landscape
	Generation	Nature generates a policy aimed at the restoration ‘original’ nature	Nature generates a form of governance aiming the restoration of the relationship with nature	Government provides subsidy for agri-environmental management by farmers to compensate for loss of production	Farmers are rewarded for agri-environmental management with a payment, biodiversity is an additional production goal
Governance and behaviour	Authority	Democratically chosen plans are meant to execute. They represent authority. Legal legitimation	Government acquires authority based on shared responsibility for natural areas	Government assumes that scientific knowledge provides legitimacy to the prescriptions	Collectives (farmer groups) are successful in recruiting farmers and in coordinating agri-environmental managements
	Agency	Involved citizens are treated as stakeholders rather than partners	Visions of nature reflect experiences in nature. Citizens are competent enough to define quality of nature and landscape. Self-organisation of landscape management is stimulated	Government assumes that financial compensation enables farmers to carry out agri-environmental management	Collectives provide a payment as well as knowledge and peer-to-peer learning
Behaviour and nature	Signification	Rules and regulations determine the access to and handling of nature	Signification is not based on rules but on beauty and local history	Biodiversity is a sign of successful governance	Biodiversity is a sign of good farming
	Identity	Residents as rightful owners of their own natural environment	Residents as part of nature	Farmers as producers	Farmers as stewards
Conclusion		Conflicts eventually led to a first step towards a more inclusive relationship between nature managers, critical residents and nature		Local ownership of landscape management and connection to ‘good farmer’ identity make room for ethics of care	

## Discussion and conclusions

In the introduction, we questioned the language of functionality, quantification and dichotomies in dominant conceptions of the relation between humans and nature. In this article, we have proposed to replace functionality with care, quantification with concern (which may include quantification) and dichotomies with relationality. We introduced a framework for landscape governance aimed at an inclusive understanding of the relations between nature, behaviour and governance. By distinguishing reciprocal dualities in mutual relation, we conceptualised landscape governance as a matter of concern, combining natality (identity, agency and generation) and creativity (signification, authority and ordering). This comprehension of landscape governance aligns better with many aspects of the frequently occurring situations where tensions arise in the system of interplay between nature, behaviour and governance, i.e. landscape.

This broader understanding could potentially contribute to new forms of governance, new behaviours and more respect for natural conditions. However, this is not an automatism. Such a framework cannot simply be turned into a checklist that only needs to be ticked to lead to success. A matter of concern approach requires an attitude, which includes both agency and authority. In practice, this requires substantial effort. Without elaborating on this here, our case studies indicate the importance of (1) acknowledgement of nature-positive identities in communication (such as stewardship or partnership), (2) collaboration with ‘caring’ local leaders, to enable caring behaviour and to constrain destructive behaviour, (3) responsiveness to different views on and different ways of dealing with nature as basis for inclusive, nature-based solutions and approaches.

However, a relational approach does not mean that all barriers and challenges simply disappear. Plurality of landscape perceptions implies that not all local actors may hold values that represent ethics of care. Opposing interests and views remain. Since governance always creates ‘winners’ and ‘losers’, governance implies choices on who and what to enable and who and what to constrain. This is an inherently political processes, which in our experience gains legitimacy when nature and local groups are seen as a matter of concern. Then concretely experienced oppositions become central, which prevents contradictions from being magnified by abstractions. For example, in spatial planning, the use of models to calculate the impact of plans—which often leads to resistance or disconnect with local stakeholders—could be embedded in forms of dialogue that give room to and acknowledge experiences.

In conclusion, the understanding of landscape and landscape governance can be deepened through a principled tripartite approach, summarised here as landscape governance triangle. The relationships between these are reciprocal and complementary. We expect that this approach can also contribute in practice to an ethics of care for nature and for people.

With proposing a relational approach, we do not support relativism or fact-free policies. One criticism of the relational approach is that it seems to lack a theoretical starting point (a view of reality) that allows selecting the best possible (efficient) options for system change (Raymond et al. 2021). In our experience, a relational approach does not deny that such a starting point is important, but combines the factuality of reality with engagement with reality. Or, to paraphrase Latour (2004): a relational approach based on concern *adds* reality to matters of fact.
